# On the Use of Auscultation and Percussion in the Diagnosis of Diseases of the Organs of Respiration and Circulation

**Published:** 1838-01

**Authors:** 


Art. IV.—*On the use of Auscultation and Percussion in the Diagnosis of Diseases of the Organs of Respiration and Circulation.* By Julius Wolff, m.d., Member of the Royal College of Göttingen, &c.—*London,* 1837. 8vo. pp. 200.

We can only account for the appearance of this work by attributing it to the author's unacquaintance with the state of our literature on the subjects of which it treats. As it contains and professes to contain nothing original, and only gives us again what we already possess in many different forms in our language, we fear its author will not be long in discovering that, like other things which are superfluous, it will neither be sought after nor valued. It is, however, due to the author to state, that his work contains a fair compendium of the present state of our knowledge of auscultation, although conveyed in a cold and unattractive manner. We wish that Dr. Wolff, instead of wasting his industry in such a supererogatory labour, had employed his time in giving to British readers—what would have been really useful to them—some of the many classical works of his countrymen which are yet untranslated.

